# Genetic factors may play a prominent role in the development of coronary heart disease dependent on important environmental factors

**DOI:** 10.1111/joim.12177

**Published:** 2014-01-03

**Authors:** C Song, Z Chang, P K E Magnusson, E Ingelsson, N L Pedersen

**Affiliations:** 1 Department of Medical Epidemiology and Biostatistics, Karolinska InstituteStockholm, Sweden; 2 Department of Medical Sciences, Molecular Epidemiology and Science for Life Laboratory, Uppsala UniversityUppsala, Sweden

**Keywords:** coronary heart disease, gene–environment interaction, heritability, modifiable lifestyle

## Abstract

**Astract:**

Song C, Chang Z, Magnusson PKE, Ingelsson E, Pedersen NL (Karolinska Institutet, Stockholm; Uppsala University, Uppsala; Sweden). Genetic factors may play a prominent role in the developmentofcoronary heart diseasedependenton important environmental factors. *J InternMed*2014; **275**: 631–639.

**Objective:**

The aim of the study was to examine whether various lifestyle factors modify genetic influences on coronary heart disease (CHD).

**Design:**

The effect of lifestyle factors [including smoking, sedentary lifestyle, alcohol intake and body mass index (BMI)] on risk of CHD was evaluated via Cox regression models in a twin study of gene–environment interaction. Using structure equation modelling, we estimated genetic variance of CHD dependent on lifestyle factors.

**Subjects:**

In total, 51 065 same-sex twins from 25 715 twin pairs born before 1958 and registered in the Swedish Twin Registry were eligible for this study. During the 40-year follow-up, 7264 incident CHD events were recorded.

**Results:**

Smoking, sedentary lifestyle and above average BMI were significantly associated with increased CHD incidence. The heritability of CHD decreased with increasing age, as well as with increasing levels of BMI, in both men and women.

**Conclusions:**

The difference in the genetic component of CHD as a function of BMI suggests that genetic factors may play a more prominent role for disease development in the absence of important environmental factors. Increased knowledge of gene–environment interactions will be important for a full understanding of the aetiology of CHD.

## Introduction

Coronary heart disease (CHD) is one of the leading causes of death worldwide 1. Previous twin studies showed moderate to high heritability of CHD mortality (40–60%) 2,3. In a recent large genome-wide association study (GWAS), it was found that 46 loci were significantly related to CHD at a genome-wide significant level, with a further 104 independent variants at a false discovery rate of 5%, which together explained 10.6% of CHD heritability 4. CHD is also highly influenced by lifestyle factors. Amongst these, smoking is one of the most well-known and established risk factors for CHD 5. A sedentary lifestyle is another major risk factor for CHD 6. It was recently shown that moderate to high levels of physical activity in leisure time had beneficial effects on CHD in a dose-dependent manner 7. The results of a systematic meta-analysis showed that all levels of alcohol consumption above 2.5 g per day reduced the risk of incident CHD, but also showed a greater risk of cardiovascular mortality and stroke incidence amongst those who consumed more than 60 g per day 8. An association has been shown between being moderately overweight or obese and an increased risk of CHD 9. Furthermore, a high body mass index (BMI) in late childhood and early adulthood (before 30 years of age) has also been related to an increased risk of later CHD 10.

Previous studies have reported several genotype–environment interaction effects on CHD. For example, smoking increased the risk of CHD in men to a greater extent in those carrying the epsilon4 allele of *APOE* 11. Further, the risk allele of *FTO* variant rs8050136 increased the risk of CHD, particularly in women who were least physically active 12. A borderline interaction effect between a genetic variant of the alcohol dehydrogenase type 3 gene and alcohol consumption on prevalent CHD has been reported 13. However, few studies have addressed the overall effects of various lifestyle factors in modifying the role of genetic effects on CHD. The aim of this study was to explore gene–lifestyle interactions on CHD using a twin design, focusing on smoking, a sedentary lifestyle, alcohol intake and BMI.

## Methods

### Study sample

In this study, we included all same-sex twins registered in the nationwide Swedish Twin Register born between 1886 and 1925 (cohort I) or between 1926 and 1958 (cohort II) 14. Information on lifestyle factors including smoking, alcohol consumption, weight, height, physical activity and other health indices was collected from questionnaires in 1961, 1963, 1967 and 1970 for cohort I and in 1973 for cohort II. Baseline age (i.e. age when answering the questionnaire) was calculated from birth year and year of questionnaire response. Individuals who were 16 years or younger when responding to the questionnaire or who died at <30 years of age were excluded from the analyses.

The study population included 51 065 individuals from 25 715 twin pairs; information was only available in one twin for 365 pairs. An indicator variable to adjust for potential cohort effects was included because of the differences in age when answering the questionnaires between the two cohorts. In cohort I, most individuals answered the questionnaire in middle to old age (45–84 years), whereas in cohort II, most individuals answered the questionnaire in young to middle age (17–47 years). Zygosity was determined based on the response to the following question: ‘During childhood, were you and your twin partner as alike as “two peas in a pod” or not more alike than siblings in general?’ 14. This method has been validated using DNA genotyping and found to be >98% accurate 14.

All participants provided informed consent, and the Ethics Committee at Karolinska Institutet approved the study protocol.

### Definition of lifestyle factors

Participants were considered as smokers if they reported having ever smoked regularly. Participants were considered to have a sedentary lifestyle if they reported ‘hardly any’ leisure-time physical activity at the age of 25–40 years in cohort I and if they reported ‘almost no’ leisure-time physical activity at the time of completing the questionnaire in cohort II. Alcohol consumption was classified as standard drinks per day based on intake of beer, wine and liquor combined (one standard drink containing 12 g ethanol 15), and values were log-transformed due to skewness. In cohort I, information was collected about height and weight at 25 years, at 40 years and at the time of completing a questionnaire in 1963, 1967 or 1970; in cohort II, information about current height and weight was collected from the questionnaire response in 1973. We used the highest recorded BMI at all these time-points to capture the lifetime likelihood of being overweight and obese. Values of the natural logarithm of BMI were *z*-score-transformed separately in men and women to standardize for easier comparison.

### Definition of the CHD outcome

Information about CHD was collected from the Swedish Patient Register and the Cause of Death Register by linkage using the unique Swedish personal identification number. All individuals were followed from the initiation of the Swedish Patient Register (in 1967; full national coverage from 1987) until their date of death or 31 December 2010. CHD cases were identified at the first occasion of a CHD diagnosis using the International Classification of Diseases (ICDs) codes: 8th revision (ICD-8) from 1968 to 1986, 410 and 411; ICD-9 from 1987 to 1996, 410 and 411; and ICD-10 from 1997 to the end of follow-up, I20, I21 and I22. Only the main diagnosis (i.e. principal cause of hospitalization or underlying cause of death) was included in the outcome definition to maximize the validity (positive predictive value ∼95%) 16,17.

### Statistical analyses

#### Lifestyle effect on CHD risk

We used Cox proportional hazards regression to estimate effect sizes of different lifestyle factors on risk of CHD, using both standard methods and within-twin pair comparisons. First, a Cox regression model was applied to assess the association between lifestyle factors and CHD, adjusting for baseline age (at the time of completing the questionnaire), sex and cohort, with a sandwich estimator to correct the standard errors for twin clustering. Secondly, Cox regression models stratified by twin pairs were applied to estimate the effects of lifestyle factors on CHD separately for monozygotic (MZ) and dizygotic (DZ) twin pairs. Only pairs discordant both for the lifestyle variables and for CHD or those with different CHD onset dates were included in these analyses. Attenuation of the association within pairs indicated that it was confounded by genetic or other familial factors. Next, stratified Cox regression including zygosity–lifestyle interaction effect in the model was applied. Significance of this interaction demonstrates that the association between lifestyle and CHD risk differs as a function of zygosity. Attenuation of the effect size in MZ pairs, compared with DZ pairs, suggests that there are shared genetic factors between the lifestyle factor and CHD. All association analyses were performed using the statistical software package stata 12.1 (StataCorp, College Station, TX, USA).

#### Lifestyle effect on genetic variance of CHD

We used a classical twin design to estimate the genetic and environmental influences on CHD variance. There are three basic assumptions about twin similarity 18: MZ twin pairs share 100% of their genes, DZ twin pairs share on average 50% of their segregating genes and all twin pairs share 100% of shared (in common) familial environment. Based on these assumptions, CHD variance can be explained in a liability threshold model by three components: additive genetic (A), shared environmental (C) and nonshared environmental (E) factors. The proportion of variance explained by additive genetic factors is also commonly termed narrow-sense heritability 18. The liability threshold model as applied to a binary variable (CHD) assumes that the ordered categories (not affected and affected) reflect an imprecise measurement of a hypothetical underlying normal distribution of liability 19. By assessing whether the genetic variance of CHD depends on certain lifestyles, insights into the possible gene–lifestyle interactions can be gained.

We first estimated the variance of CHD explained by A, C and E components. We then tested nested AE models (excluding C) using likelihood ratio tests. As decided *a priori*, if the AE model was not significantly different from the full ACE model (i.e. the shared environmental component C was not significant), AE models were used in further analyses. Models were adjusted for age at baseline and cohort.

Lifestyle factors could affect CHD risk via interactions with genetic factors, and potential interactions would be demonstrated by differential genetic variance of CHD as a function of certain lifestyles. A moderator model for gene–environment interaction was first proposed by Purcell 20 for continuous data, and extensions are available for binary data 21,22. That is, the variance of the binary variable (i.e. CHD) is constrained to unity at a specified value of the moderator (e.g. the mean), and the variance is allowed to differ across levels of the moderator above and below the mean 21. The lifestyle factors were evaluated as putative moderators of AE variance components for CHD (Figure S1) using these models. The path parameters *x* (A component) and *z* (E component) can be moderated by lifestyle factors according to the equations: (*x* + β_a_*M) and (*z* + β_e_*M), respectively, where *M* is the moderator, and β_a_ and β_e_ refer to the moderating effects on A and E components, respectively. Genetic effects shared between CHD and the investigated lifestyle factors were removed by adjusting for the effect of lifestyle factors on CHD risk through β_*M*1_*M1 and β_*M*2_*M2, where β_*M*1_ and β_*M*2_ are the coefficients of the moderating effect in twin 1 and twin 2, respectively (see Figure S1). β_a_ represents the modifying effect of lifestyle on genetic variance of CHD (after taking into account the genetic effects of that particular lifestyle factor). A moderating effect of lifestyle on genetic variance of CHD was evident when β_a_ was significantly different from zero. Because heritability of CHD death was reported to be lower in older age groups 23, we also used the moderator model to estimate the difference in variance components across different age groups. When the moderator was normally distributed (i.e. age and log-transformed BMI), we used a *z*-score to standardize the moderator and variance in CHD as a function of SD units of the moderator. Thus, the β_a_ and β_e_ values represent the changes in A and E path parameters per 1 SD change in the moderator, respectively.

If a lifestyle factor showed evidence of a significant moderating effect on CHD genetic variance, we further included both age at baseline and lifestyle as two moderators in the same model, to investigate the moderating effect of lifestyle factors on genetic variance of CHD after controlling for age.

All structural equation modelling was performed using maximum-likelihood model fitting with raw data in the program Mx 1.703 24.

## Results

There were 7264 incident CHD cases amongst the 51 065 individuals from 25 715 twin pairs; 4965 twin pairs were discordant for CHD and 1119 twin pairs both had CHD, and 61 pairs with CHD information from only one twin. Baseline characteristics including age and lifestyle factors are shown in Table1. Participants in cohort I were on average 30 years older than those in cohort II when answering the questionnaire. CHD prevalence was lower in women and age of onset of disease was significantly higher in women than in men. Compared with men, women smoked less, consumed less alcohol, were more likely to have a sedentary lifestyle and had a slightly lower mean BMI (*P *<* *0.001 for all comparisons). Because CHD prevalence and onset age as well as lifestyle factors all differ according to gender, all results below are presented separately for men and women.

**Table 1:** Characteristics of the study sample at baseline

Baseline characteristics	Men (*n *=* *23 692)	Women (*n *=* *27 373)
Age when answering the questionnaire, years	41.3 ± 16.7	43.0 ± 17.1
Cohort I (*n *=* *30 212)	59.0 ± 9.8	59.6 ± 10.1
Cohort II (*n* = 20 853)	30.2 ± 8.6	30.7 ± 8.6
Age of CHD onset (only for CHD cases)	69.2 ± 11.1	75.4 ± 10.6
Cohort I (*n *=* *4657)	74.5 ± 9.0	79.2 ± 8.3
Cohort II (*n *=* *2607)	61.9 ± 9.4	65.4 ± 9.5
Number of CHD cases	4258	3006
Regular smoker, % (*n *=* *46 702^*^)	54.1	33.7
Pack-years of smoking (only among smokers)	9.5 ± 9.7	6.7 ± 6.7
Sedentary lifestyle, % (*n *=* *43 411^*^)	11.2	12.6
Alcohol consumption, standard drinks/day (*n *=* *38, 142^*^)	0.75 ± 1.00	0.23 ± 0.41
Standard drinks/day (only among drinkers)	0.93 ± 1.03	0.43 ± 0.49
Body mass index, kg m^−2^ (*n *=* *45 746^*^)	23.9 ± 3.2	23.2 ± 4.0

Study characteristics are given for participants at the time of completing the questionnaire. Continuous variables are presented as mean ± standard deviation, and binary variables as percentage.

One pack-year is equivalent to smoking 20 cigarettes per day for 1 year.

One standard drink contains 12 g ethanol.

### Heritability of CHD

The proportions of additive genetic (A), shared environmental (C) and nonshared environmental (E) variance for CHD risk adjusted for age and cohort are shown in Table2. We found that shared environment accounted for very little CHD variance (nonsignificant effect) in both men and women. Therefore, we used only AE models in further analyses.

**Table 2:** Genetic and environmental components of variance for CHD in men and women

	Proportion of variance (95% CI)			Fit of model[Table-fn tf2-2]		
Additive genetic	Shared environment	Nonshared environment	−2LL	Δχ^2^ (df)	*P* value
Men						
ACE model	0.37 (0.24, 0.51)	0.10 (0, 0.19)	0.54 (0.48, 0.59)	20782.57	–	–
AE model	0.48 (0.44, 0.53)	–	0.51 (0.47, 0.56)	20785.40	2.83 (1)	0.092
Women						
ACE model	0.36 (0.19, 0.36)	0.01 (0, 0.14)	0.63 (0.63, 0.68)	17381.60	–	–
AE model	0.37 (0.35, 0.37)	–	0.63 (0.63, 0.64)	17381.64	0.04 (1)	0.839

−2LL, −2 log likelihood; df, degrees of freedom; Δχ^2^, change in chi-square; A, additive genetic component; C, shared environment component; E, nonshared environment component; CI, confidence interval.

* Model fitting comparison when shared environment component C is removed.

**Figure 1:**
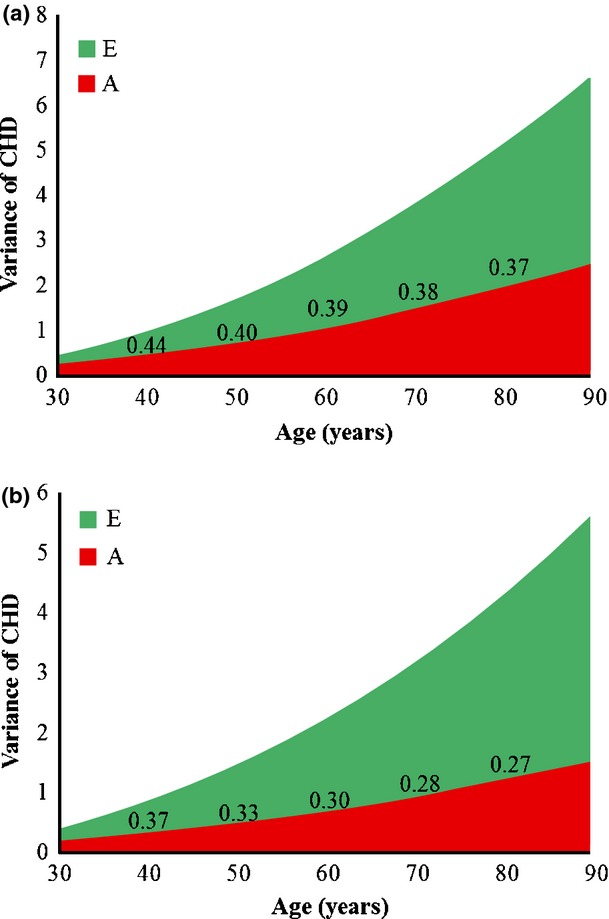
Genetic variance (A), nonshared environmental variance (E) and heritability of CHD as a function of age in men (a) and women (b). Each unit on the *x*-*axis represents age. Heritability of CHD*, *as a proportion of the total variance, is shown in the figure for selected ages (40, 50, 60, 70 and 80* *years)*. *CHD*, *coronary heart disease*.

Next, we investigated whether age moderated the AE components of CHD variance. Both genetic and nonshared environmental variance components increased across the age groups (Fig.1). However, the increase in total variance across age groups was explained by greater increases in environmental variance. Thus, expressed as a proportion of total variance, heritability decreased with increasing age.

### Smoking and CHD

Smoking significantly increased the risk of CHD in both men and women, and the effect did not differ substantially within MZ and DZ pairs (Table3). The variance explained by the genetic component did not significantly differ between smokers and nonsmokers in either men or women (Table4). Heritability of CHD in men and women was 0.56 and 0.36 in nonsmokers and 0.44 and 0.41 in smokers, respectively. Heritability differed between smokers and nonsmokers as a result of a significant effect of smoking on the environment component (Table S1). Thus, our results confirm that smoking is a risk factor for CHD in both men and women, but we found no evidence of a smoking–gene interaction.

**Table 3:** Associations between lifestyle factors and CHD risk adjusted for age and cohort

Lifestyle factor	Sex	Cox regression correcting for relatedness		Stratified Cox regression in MZ pairs		Stratified Cox regression in DZ pairs		Zygosity–lifestyle interaction
		HR (95% CI)	*P* value	HR (95% CI)	*P* value	HR (95% CI)	*P* value	*P* value
Smoking	Men	1.27 (1.19, 1.36)	<0.001	1.16 (0.86, 1.57)	0.323	1.18 (0.99, 1.41)	0.066	0.930
		Women	1.37 (1.26, 1.50)	<0.001	1.77 (1.23, 2.53)	0.002	1.61 (1.27, 2.05)	<0.001	0.683
Sedentary lifestyle	Men	1.09 (0.98, 1.22)	0.099	1.48 (1.01, 2.17)	0.046	0.94 (0.73, 1.20)	0.609	0.051
		Women	1.14 (1.01, 1.28)	0.033	1.36 (0.92, 1.99)	0.122	1.140.84, 1.55	0.395	0.491
Alcohol consumption	Men	0.99 (0.91, 1.08)	0.842	0.95 (0.67, 1.35)	0.772	0.95 (0.76, 1.19)	0.654	0.997
		Women	0.89 (0.74, 1.08)	0.248	0.74 (0.36, 1.51)	0.410	1.49 (0.93, 2.37)	0.094	0.108
BMI	Men	1.20 (1.15, 1.24)	<0.001	1.26 (1.05, 1.50)	0.011	1.15 (1.03, 1.27)	0.01	0.372
		Women	1.20 (1.15, 1.26)	<0.001	1.21 (0.93, 1.57)	0.155	1.08 (0.95, 1.22)	0.227	0.437

HR, hazard ratio; CI, confidence interval; MZ, monozygotic; DZ, dizygotic; BMI, body mass index.

Zygosity was classified as MZ = 1, DZ = 2 in the zygosity–lifestyle interaction term.

**Table 4:** Age and lifestyle moderating effects (β_a_ and β_e_) on variance components of CHD

	Men		Women	
Moderating effect on genetic component (95% CI)	Moderating effect on environmental component (95% CI)	Moderating effect on genetic component (95% CI)	Moderating effect on environmental component (95% CI)
Age	0.31 (0.27, 0.31)	0.45 (0.41, 0.46)	0.23 (0.19, 0.24)	0.45 (0.37, 0.45)
Smoking	0.06 (−0.06, 0.19)	0.26 (0.12, 0.40)	−0.12 (−0.24, 0.01)	−0.22 (−0.32, −0.10)
Sedentary lifestyle	0.03 (−0.15, 0.25)	−0.10 (−0.29, 0.12)	0.24 (−0.01, 0.53)	0.01 (−0.24, 0.30)
Alcohol consumption	−0.15 (−0.27, −0.01)	−0.08 (−0.22, 0.07)	0.12 (−0.32, 0.36)	−0.34 (−0.55, 0.06)
BMI	0.07 (0.03, 0.11)	0.26 (0.20, 0.33)	0.05 (0.00, 0.09)	0.33 (0.24, 0.38)

CI, confidence interval; BMI, body mass index.

Moderating effect on genetic component is β_a_, and moderating effect on environmental component is β_e._

### Sedentary lifestyle and CHD

Sedentary lifestyle was associated with an increased risk of CHD in women, with a similar trend in men; there was no attenuation of the association in the within-twin pair analyses (Table3). There was no significant moderating effect of a sedentary lifestyle on the genetic variance component (Table4), thus providing little evidence of a gene–sedentary lifestyle interaction on CHD.

### Alcohol consumption and CHD

Alcohol consumption as a continuous measure (drinks per day) was not associated with CHD risk (Table3). When comparing abstainers (no alcohol consumption at all) versus ever-drinkers, we observed a lower risk of CHD in ever-drinkers amongst women (Table S2), but not in the within-pair analysis. Genetic variance of CHD in men differed as a function of alcohol consumption with borderline significance (*P *=* *0.033; Tables4 and S1). Heritability of CHD in men who consumed no alcohol was 0.47 and lower (0.41) amongst those who consumed 2.7 standard drinks per day.

### BMI and CHD

Higher BMI was associated with greater risk of CHD; this association was not attenuated within MZ or DZ twin pairs (Table3). Both genetic and nonshared environmental variance of CHD significantly differed as a function of BMI (Tables4 and S1) in men and women. Heritability of CHD also decreased with increasing levels of BMI. These results indicate the presence of a BMI–gene interaction effect on CHD.

**Figure 2:**
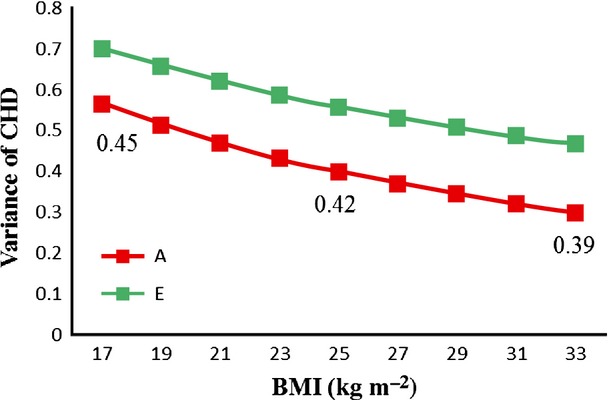
Variance components of CHD as a function of BMI in men. Genetic (A) and nonshared environmental (E) variance components of CHD versus BMI at the mean age at baseline are shown. Heritability of CHD, as a proportion of the total variance, is shown for BMI values of 17, 25 and 33 kg m^−2^. BMI, body mass index; CHD, coronary heart disease.

Because BMI is correlated with age, which also moderates genetic variance of CHD (Fig.1 and Table4), we performed additional analyses allowing both age and BMI to moderate the AE components of CHD variance. Again, both genetic and nonshared environmental variance of CHD were greater in older age (Table S3) and lower with higher BMI (statistically significant only in men). Heritability of CHD decreased with increasing BMI. The effect of BMI on AE components and heritability at the mean baseline age (40 years) in men is shown in Fig.2. The same pattern was observed across the age groups (data not shown).

## Discussion

In this large study of 51 065 individuals from 25 715 twin pairs with 7264 incident CHD events, we confirmed well-known associations of smoking, sedentary lifestyle and increased BMI with increased CHD risk. We found that the heritability of CHD incidence decreased with increasing age. Furthermore, after controlling for age, the genetic variance of CHD decreased with higher BMI in men and showed the same trend in women, indicating a BMI–gene interaction effect on the incidence of CHD. We also detected a borderline significant effect on CHD incidence of an alcohol consumption–gene interaction in men.

This was the first study to demonstrate a moderating effect of BMI on genetic variance of CHD incidence after controlling for age. The heritability of CHD decreased with higher BMI. Thus, genetic factors may play a more important role for development of CHD in individuals with a low compared with a high BMI, through two possible underlying mechanisms: (i) additional genes may influence CHD variance in those with a low BMI, and (ii) the magnitude of the effect of some CHD-related genes may differ by level of BMI. To our knowledge, no gene–environment interaction in CHD GWASs has been reported. However, it was reported that an HDL-related genetic score based on previous GWASs had a smaller effect on HDL levels with increasing BMI 25. Thus, these findings are in agreement with our results, potentially suggesting higher gene expression of HDL- or CHD-related genes in lean compared with obese individuals. Further GWASs to address gene–BMI interactions should be conducted, as our findings indicate that such studies could identify additional CHD susceptibility variants specific for different levels of BMI. This may be especially beneficial in the normal-weight population as these susceptibility variants may be masked in GWASs including individuals with higher BMI.

We also found that genetic variance as well as the heritability of CHD tended to be lower in men consuming more alcohol. As with BMI, this could indicate higher expression of CHD-related genes in individuals who consume low to moderate alcohol levels compared with the high alcohol consumption group. However, these borderline significant results should be interpreted with caution, and an independent study with a larger sample size is required to robustly confirm this alcohol consumption–gene interaction effect on CHD.

Our results showed that genetic variance of CHD incidence increases with age, but not as much as nonshared environmental variance. Thus, the heritability of CHD was lower in older age groups. This is consistent with the finding of a previous study, based on data from the Swedish Twin Registry, that heritability of CHD mortality decreased with increasing age 23. Other findings from the same group indicated that several risk factors (including smoking, BMI, hypertension, diabetes, marital status, level of education and birth cohort) together only contribute to the environmental component of variance for CHD mortality 3. Our findings extend these previous results, giving new important insights into the nature of genetic variance for CHD by showing that different lifestyle factors, including smoking and a sedentary lifestyle, increase the risk of CHD directly rather than through interaction with genetic factors.

Heritability of CHD represents the relative importance of genes versus the environment. Differences in heritability reflect changes in genetic or environmental risk factors or both. However, when exploring gene–lifestyle interactions, differences in heritability are evident only when the genetic component of CHD variance differs as a function of lifestyle factors. Hence, heritability differences could be masked or caused by differences in the environmental component. For example, heritability of CHD differed between smokers and nonsmokers, due to nonshared environmental differences.

Purcell and colleagues have noted that in twin modelling studies, the effect of a moderator could reflect either (i) lifestyle moderating the effects of certain genes (gene–environment interaction), or (ii) ‘trait-influencing genes being more likely to be present in that environment’ (gene–environment correlation) 20. In the present study, the associations between lifestyle and CHD were not attenuated within-twin pairs, which suggests that there is little evidence of a gene–lifestyle correlation. Further, when estimating the contribution of lifestyle as a moderator of ACE variance components for CHD, we adjusted for lifestyle on risk of CHD. Thus, the potential genetic correlation between CHD and lifestyle was removed from these analyses 20,22. By modelling in this way, the effects of genes that may be shared by CHD and lifestyle are eliminated, providing the true lifestyle–gene interaction 26.

The strengths of our study include the very large sample with many incident CHD events and up to 40 years of follow-up. To our knowledge, this is the largest twin study so far to evaluate gene–lifestyle interaction effects on CHD. This study also has several limitations. First, lifestyle factors are time- and age-dependent exposures. Because we included data from only one questionnaire completed at the time of study entry, we do not know how lifestyle varied during the follow-up, leading to some degree of misclassification bias. Secondly, as the study was based on self-reported lifestyle behaviour, there is a potential for under-reporting of smoking and alcohol consumption and over-reporting of exercise, which could bias our results towards the null. Thirdly, as this study was performed in individuals of Northern European descent, the generalizability to other ethnic groups is unknown.

In summary, in this nationwide twin study of 51 065 individuals, we demonstrated that higher BMI was associated with lower genetic variance of CHD indicating that genetic factors play a more prominent role for disease development in the absence of important environmental factors. Our results suggest that further insight into the effects of gene–environment interactions would improve understanding of the aetiology of CHD.

## Conflict of interest statement

None of the authors has any potential conflict of interests.

## Supporting Information

Table S1 Model fitting parameters for successively reduced models to test the effects of lifestyle factors on A and E components of coronary heart disease (CHD) variance. **Table S2.** Effect of being ever-drinker on coronary heart disease (CHD) risk compared with being abstainer, adjusted for age and cohort. **Table S3.** Moderating effects of age and BMI on additive genetic component of coronary heart disease (CHD). **Figure S1.** Moderator twin model for gene–environment interaction.
