# Supplementary material for: Genetic factors may play a prominent role in the development of coronary heart disease dependent on important environmental factors
Source: J Intern Med. 2014 Jan 3;275(6):631–9. doi: 10.1111/joim.12177 (PMC4288985; doi:10.1111/joim.12177)
Supplement: Table S1 — Model fitting parameters for successively reduced models to test the effects of lifestyle factors on A and E components of coronary heart disease (CHD) variance. Table S2. Effect of being ever-drinker on coronary heart disease (CHD) risk compared with being abstainer, adjusted for age and cohort. Table S3. Moderating effects of age and BMI on additive genetic component of coronary heart disease (CHD). Figure S1. Moderator twin model for gene–environment interaction. [file joim0275-0631-sd1.doc]

**Supplementary Table 1** Model fitting parameters for successively reduced models to test the effects of lifestyle factors on A and E components of coronary heart disease (CHD) variance

| Models | Model fitting parameters | | | | | |
| --- | --- | --- | --- | --- | --- | --- |
|  | Men | | | Women | | |
|  | -2LL | df | *P* value (∆df) | -2LL | df | *P* value (∆df) |
| Full model dependent on smoking | 17,252.53 | 20,415 |  | 14,699.52 | 24,377 |  |
| 1)      No moderating effect on A (βa = 0) | 17,253.37 | 20,416 | 0.841 (1) | 14,702.78 | 24,378 | 0.071 (1) |
| 2)      No moderating effect on E (βe = 0) | 17,267.94 | 20,416 | <0.001 (1) | 14,712.19 | 24,378 | <0.001 (1) |
| 3)      No moderating effect on A or E (βa = 0, βe = 0) | 17,268.15 | 20,417 | <0.001 (2) | 14,719.07 | 24,379 | <0.001 (2) |
|  |  |  |  |  |  |  |
| Full model dependent on sedentary lifestyle | 15,634.59 | 17,755 |  | 13,590.79 | 21,783 |  |
| 1)      No moderating effect on A (βa = 0) | 15,634.70 | 17,756 | 0.744 (1) | 13,594.32 | 21,784 | 0.060 (1) |
| 2)      No moderating effect on E (βe = 0) | 15,635.41 | 17,756 | 0.365 (1) | 13,590.80 | 21,784 | 0.930 (1) |
| 3)      No moderating effect on A or E (βa = 0, βe = 0) | 15,635.46 | 17,757 | 0.646 (2) | 13,595.45 | 21,785 | 0.097 (2) |
|  |  |  |  |  |  |  |
| Full model dependent on alcohol consumption | 14,930.35 | 15,419 |  | 13,273.42 | 18,805 |  |
| 1) No moderating effect on A (βa = 0) | 14,934.90 | 15,420 | 0.033 (1) | 13,274.58 | 18,806 | 0.281 (1) |
| 2) No moderating effect on E (βe = 0) | 14,931.57 | 15,420 | 0.274 (1) | 13,276.46 | 18,806 | 0.081 (1) |
| 3) No moderating effect on A or E (βa = 0, βe = 0) | 14,935.48 | 15,421 | 0.077 (2) | 13,276.66 | 18,807 | 0.198 (2) |
|  |  |  |  |  |  |  |
| Full model dependent on BMI | 17,251.22 | 19,287 |  | 15,000.98 | 23,491 |  |
| 1)      No moderating effect on A (βa = 0) | 17,260.68 | 19,288 | 0.002 (1) | 15,004.84 | 23,492 | 0.049 (1) |
| 2)      No moderating effect on E (βe = 0) | 17,317.41 | 19,288 | <0.001 (1) | 15,042.27 | 23,492 | <0.001 (1) |
| 3)      No moderating effect on A or E (βa = 0, βe = 0) | 17,328.18 | 19,289 | <0.001 (2) | 15,056.27 | 23,493 | <0.001 (2) |

-2LL and df refer to the model listed; *P* value and ∆df refer to the change in log-likelihood when comparing sub-models to the full model.

β_a_ refers to the function of moderating effect on genetic component (A) of CHD variance; β_e_ refers to the function of moderating effect on non-shared environmental component (E) of CHD variance.

CHD liability was adjusted for baseline age, cohort and the lifestyle factor.

-2LL, -2 log likelihood; df, degrees of freedom; ∆df, change in degrees of freedom; A, additive genetic component; E, non-shared environment component; BMI, body mass index.

**Supplementary Table 2 Effect of being ever-drinker on coronary heart disease (CHD) risk compared with being abstainer, adjusted for age and cohort**

| **Lifestyle factor** | **Sex** | **Cox regression correcting for relatedness** | | **Stratified Cox regression in MZ pairs** | | **Stratified Cox regression in DZ pairs** | | **Zygosity by lifestyle interaction** |
| --- | --- | --- | --- | --- | --- | --- | --- | --- |
|  |  |  |  |  |  |  |  |  |
|  |  | **HR** | ***P* value** | **HR** | ***P* value** | **HR** | ***P* value** | ***P* value** |
|  |  | **(95% CI)** |  | **(95% CI)** |  | **(95% CI)** |  |  |
| **Ever-drinker** | Male | 0.99 | 0.868 | 0.82 | 0.253 | 0.90 | 0.359 | 0.646 |
|  |  | (0.91, 1.08) |  | (0.58, 1.16) |  | (0.72, 1.13) |  |  |
|  | Female | 0.90 | 0.010 | 1.07 | 0.678 | 0.99 | 0.913 | 0.650 |
|  |  | (0.83, 0.98) |  | (0.77, 1.48) |  | (0.80, 1.23) |  |  |

HR, hazard ratio; CI, confidence interval; MZ, monozygotic; DZ, dizygotic.

Zygosity was classified as MZ=1, DZ=2 in the zygostiy–lifestyle interaction term.

**Supplementary Table 3 Moderating effects of age and BMI on additive genetic component of coronary heart disease (CHD)**

|  | **Moderating effect on additive genetic component of CHD (β_a_)** | |
| --- | --- | --- |
|  | **Age**  **(95% CI)** | **BMI**  **(95% CI)** |
| **Men** | 0.30  (0.25, 0.31) | -0.04  (-0.07, 0.00) |
| **Women** | 0.24  (0.19, 0.24) | -0.06  (-0.11, 0.02) |

BMI, body mass index; CI, confidence interval.

Supplementary Figure legends

**Supplementary Fig. 1** Moderator twin model for gene–environment interaction. The moderator (M), which is age or lifestyle factors in this study, could affect both coronary heart disease (CHD) risk and CHD variance via genetic (A) and non-shared environmental (E) components. The path parameter A (x) and E (z) components can be moderated by the moderator (M) according to (x + β_a_*M) and (z + β_e_*M), respectively. β_a_ and β_e_ are the coefficients of moderating effect on A and E, respectively. The risk of CHD (p) was adjusted by age (β_age_), cohort effect (β_cohort_) and the moderator effect in both twin 1 and twin 2 (β_m1_ and β_m2_, respectively).

**Supplementary Figure 1. Moderator twin model for gene x environment interaction**

**
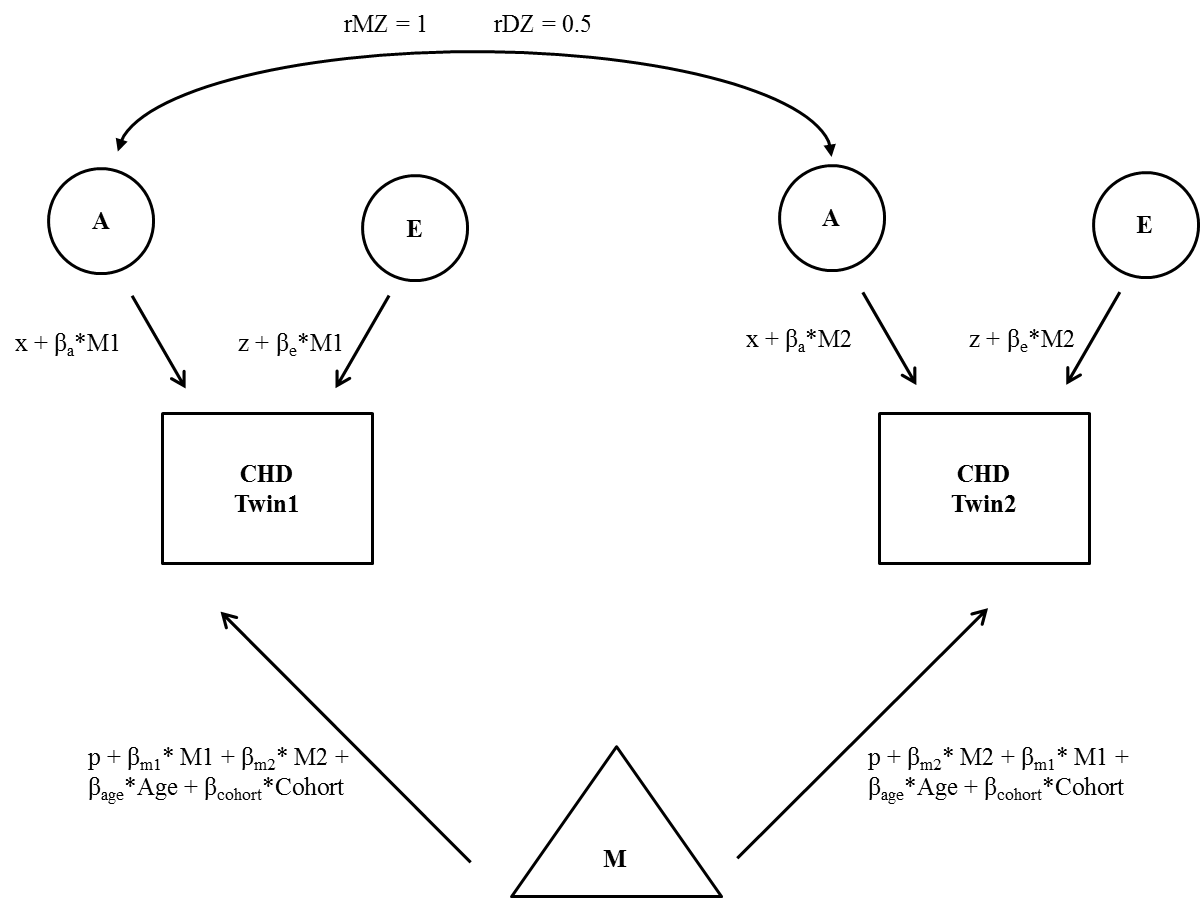
**
